# Mindfulness-Based Intervention for Adolescents with Recurrent Headaches: A Pilot Feasibility Study

**DOI:** 10.1155/2015/508958

**Published:** 2015-12-22

**Authors:** Toni Hesse, Laura G. Holmes, Vicki Kennedy-Overfelt, Lynne M. Kerr, Lisa L. Giles

**Affiliations:** ^1^Department of Psychiatry, University of Utah, Salt Lake City, UT 84108, USA; ^2^Department of Psychology, University of Utah, Salt Lake City, UT 84112, USA; ^3^Mindfulness Utah LLC, Salt Lake City, UT, USA; ^4^Department of Pediatrics, Division of Pediatric Neurology, University of Utah, Salt Lake City, UT 84108, USA; ^5^Department of Pediatrics, Division of Pediatric Psychiatry, University of Utah, Salt Lake City, UT 84108, USA

## Abstract

Recurrent headaches cause significant burden for adolescents and their families. Mindfulness-based interventions (MBIs) have been shown to reduce stress and alter the experience of pain, reduce pain burden, and improve quality of life. Research indicates that MBIs can benefit adults with chronic pain conditions including headaches. A pilot nonrandomized clinical trial was conducted with 20 adolescent females with recurrent headaches. Median class attendance was 7 of 8 total sessions; average class attendance was 6.10 ± 2.6. Adherence to home practice was good, with participants reporting an average of 4.69 (SD = 1.84) of 6 practices per week. Five participants dropped out for reasons not inherent to the group (e.g., extracurricular scheduling); no adverse events were reported. Parents reported improved quality of life and physical functioning for their child. Adolescent participants reported improved depression symptoms and improved ability to accept their pain rather than trying to control it. MBIs appear safe and feasible for adolescents with recurrent headaches. Although participants did not report decreased frequency or severity of headache following treatment, the treatment had a beneficial effect for depression, quality of life, and acceptance of pain and represents a promising adjunct treatment for adolescents with recurrent headaches.

## 1. Introduction

Headaches in pediatric patients are very common. Approximately 60% of children and adolescents have headaches over the course of their youth [1] and 20% report frequent or severe headaches in the past 12 months [2]. Headaches are a common cause of school absences, doctor visits, and analgesic medication overuse, are associated with comorbid depression and anxiety, and have adverse impact on quality of life [3–5]. This pattern of recurrent headaches creates a burden that “not only is a nuisance for some individuals but also entails widespread suffering and loss of opportunities for patients and their families and large cost for society” [6]. Common treatments of recurrent headaches include life style modification (e.g., dietary restrictions and sleep hygiene), analgesic medications, rescue/abortive medication, preventative/prophylactic medications, complementary and alternative medicines, and psychotherapeutic interventions [3, 5]. Despite these many treatment modalities, recurrent headaches persist, indicating a need for alternative treatment approaches.

Mindfulness-based interventions (MBIs) are a growing field of group-based, psychoeducational interventions with large potential application to recurrent headaches in pediatric patients. MBIs include mindfulness-based stress reduction (MBSR) and mindfulness-based cognitive therapy (MBCT). Further, many adaptations have been developed to accommodate specific populations (e.g., adolescents). A common pillar of MBIs is the practice of mindfulness through “paying attention in a particular way: on purpose, in the present moment, and nonjudgmentally” [7]. This results in a skilled ability to bring attention and awareness to an experience with openness and acceptance, so as to experience on a moment-to-moment basis rather than ruminating on the past or worrying about the future [7]. When attention is achieved in this manner, a person is able to experience events, thoughts, and emotions without becoming immersed or overwhelmed and is able to have a more balanced sense of emotions [7–9]. Furthermore, a goal of MBIs is to teach participants “acceptance” such that one is able to be aware of thoughts, emotions, and body sensations without judgment or evaluation [8]. This is achieved through decentering, emphasizing observation over judgment, and describing sensations rather than attempting to change these experiences [10]. This refined awareness is postulated to allow chronic pain sufferers to alter the experience of pain and perhaps improve ability to cope [9]. MBIs are structured through use of manuals which often include weekly group sessions, lasting from 1 to 2.5 hours each, for 8 to 12 weeks. During each session, group facilitators teach participants about the science and practice of mindfulness, provide modeling, rehearsal, and feedback as participants learn core mindfulness practices (e.g., body scan, yoga, sitting, and walking meditation), and offer participants a supportive environment in which to learn. Additionally, MBIs invite participants to engage in 20–60 minutes of daily practice of mindfulness exercises that were taught during the weekly session.

Researchers have been increasingly interested in the beneficial effects of MBIs over the past decade. Among adults, researchers have examined MBIs in treatment of anxiety, depression, cardiovascular disease, cancer, immunity, psoriasis, type 2 diabetes, and sleep disturbances [9, 11, 12]. Additionally, the effect of MBIs has been examined in chronic pain conditions such as back/neck pain, arthritis, and fibromyalgia, with varying degrees of improvement on quality of life indices [13]. MBI use for chronic headaches in adults resulted in improved sense of control of headache pain [14]. Furthermore, a recent pilot study (*N* = 19) found MBSR to be a safe and feasible adjunct to pharmacological treatment for adults with chronic headaches [15]. Although not powered to detect changes in headache frequency or severity, the study found that MBSR reduced headache duration and headache disability and improved self-efficacy for managing headache-related pain.

In contrast to the growth of research on MBIs for adults, the feasibility and efficacy of MBIs for the pediatric population have been less well established. Researchers have begun to examine the utility of MBI in treatment of pediatric depression, anxiety, ADHD, and emotion regulation, with limited yet encouraging results. In children with anxiety or high stress, MBCT was “effective in reducing attention related problems and showed promise in managing anxiety symptoms and behavior problems” [10].

Previous studies have demonstrated efficacy in treatment of recurrent pediatric headaches with psychotherapies such as cognitive behavioral therapy and biofeedback [5, 16, 17]. Additionally, preliminary research indicates that acceptance and commitment therapy and hypnosis may also reduce headache frequency, duration, and related impairment [5]. MBIs enable participants to engage in life on a moment-to-moment basis, experiencing negative events without becoming overly engaged with them [7]. This may allow adolescents who suffer with recurrent headaches to alter the experience of pain and thereby improve their ability to cope [9]. Given the burden of headaches in pediatric patients and the growing research showing benefit of MBIs for chronic headaches in adults, MBI may be a beneficial adjunct treatment for pediatric headaches.

We conducted a pilot study to demonstrate that a MBI specifically adapted for adolescents is a feasible treatment for patients with recurrent headaches. Our primary aim was to determine whether a MBI treatment was feasible for adolescent girls with recurrent headaches and specifically whether participants would attend the majority of sessions and would report doing the assigned mindfulness practices at home between sessions. Our secondary aim was to determine whether treatment with a MBI would reduce headache burden and improve participant/parent-reported quality of life.

## 2. Methods

### 2.1. Study Population

Participants were recruited via referrals from an academic pediatric neurology clinic, academic general pediatric clinic, and other community primary care clinics from November 2013 to January 2014. Patients at the clinics were prescreened for inclusion criteria during their routine scheduled visit and were provided with information about the study. The parents of potential adolescent participants provided their contact information and were evaluated via a telephone screen to determine whether their child met inclusion criteria for the study (*n* = 54). Adolescents were eligible to participate in the study if they had recurrent headaches, defined as four or more headaches per month occurring over at least three months prior to study onset. Exclusion criteria included (a) diagnosed developmental delay, intellectual disability, or autism spectrum disorder as this might limit appropriate participation in the group setting and (b) significantly abnormal neurological examination, imaging studies, laboratory studies, or structural brain pathology that contributed directly to headaches. Of the initial 54 participants who indicated interest during their routine visit, 14 declined to participate (e.g., due to travel, academic schedule, time, and disinterest in group therapy) and 13 could not be reached. Of the 27 who indicated interest in the study, 6 did not come to the first session and one parent attended the first session without her daughter and was excluded from participation; 20 were enrolled in the study. Participants were provided the MBI intervention for free; no other inducements to participate were provided.

### 2.2. Study Design

For this Institutional Review Board-approved initial pilot study, a nonrandomized clinical trial was conducted to determine the feasibility of providing MBI group treatment. Participants were allowed to continue taking prophylactic or abortive medications as prescribed and to continue other treatments as usual (e.g., dietary modifications, routine neurological or primary care visits). The treatment was conducted in a children's hospital, and participant safety was assured by having clinical psychologists and a psychiatrist present during all treatment sessions. This pilot study was not funded and there were no conflicts of interest to report.

### 2.3. Mindfulness-Based Intervention

To our knowledge, there is currently no published mindfulness group intervention manual for adolescent outpatient treatment. We therefore adapted our intervention from the Mindful Schools curriculum for adolescents (http://www.mindfulschools.org/), which was created by Mindful Schools personnel for use with groups of adolescents in a school setting for eighteen 15-minute lessons [18]. We adapted this format for the clinical setting by combining the lessons into eight 2-hour sessions delivered weekly in the evening. Sessions were led by three instructors who had received training from Mindful Schools (LGH, VKO, and a graduate student in psychology) and one psychiatrist (TH) who had previously attended a course of MBSR; all instructors maintained their own MBSR practice before and during group facilitation. The primary instructor (VKO), a certified MBSR instructor, was trained at the Center for Mindfulness in Medicine, Health Care, and Society at the University of Massachusetts Medical School, where Dr. Jon Kabat-Zinn originally developed the MBSR intervention. In addition, this instructor had 9 years of supervised MBSR group leadership training.

The intervention was purposefully minimally tailored to address headaches. During the initial session, participants were presented with the research evidence that this treatment could benefit headaches and related distress. Once treatment began, group facilitators did not provide concrete didactic education on how the mindfulness lessons would help alleviate headaches or related distress. Instead, the facilitators allowed the students to initiate discussion of headaches, anxiety, and depression themselves during group discussions. Participants were encouraged to be open to various ways that the treatment could be beneficial beyond or instead of reducing severity or frequency of headaches. One important modification designed to reduce interpersonal conflict, improve group cohesion, and normalize the experiences of participants was sharing headache triggers. During the second session, participants anonymously provided their headache triggers (e.g., lotion or body odor, loud noises, and certain lights) to facilitators, who read them aloud and asked the group to avoid triggers when possible.

Full class descriptions and outlines are available from the corresponding author. In general, sessions were structured with homework and incentives in the beginning, followed by a “welcoming and centering” practice (e.g., awareness of breath, mindfulness of sound), two 15-minute didactic lessons involving group discussion, and a 10–15-minute “food for thought” break in which participants discussed how quotes or poems related to their experiences in small groups while having a snack, followed by another 15-minute didactic lesson. Finally, there was a 10-minute discussion of home practice for the week and a closing mindfulness practice. Throughout each session there were one or two opportunities for participants to write in a journal. Journaling prompts were adapted from the Mindful Schools curriculum (e.g., What kind of thoughts arose during your practice? What have you learned about emotions over the past several lessons? Do you spend more time thinking about the past or the future?). Participants were given small incentives (e.g., colored pens, chap stick) for returning their daily diaries.

For home practice, participants were instructed to listen to a 10–15-minute guided meditation as often as possible, and at least once per day. They were provided with awareness of breath, heartfulness, and body scan guided meditations and learned mindful listening, eating, and walking during class. For the last two weeks, participants were encouraged to practice the guided meditation of their choice or to practice in silence. Participants were also asked to complete daily diaries that included formal practice, informal practice (e.g., noticing, doing tasks mindfully), headache frequency, and severity and whether their headache had interfered with daily living activities.

### 2.4. Outcome Measures

The primary outcome was the feasibility of recruiting and maintaining a group of adolescents willing to attend groups and engage in daily mindfulness practice. Secondary outcomes included change in headache-related disability/impact, anxiety, depression, and quality of life and change in headache frequency and severity (as measured by daily diaries). Baseline measures were completed at the first meeting (Week 1).

#### 2.4.1. Daily Diary

Participants maintained daily paper logs of formal mindfulness practices, informal mindfulness practices, and headaches (number of days headache is present, presence of severe headache, mild headache, or no headache). Starting on Week 2, participants were asked to complete diaries for the six days between attending each weekly session. Participants noted whether they engaged in formal and informal mindfulness practice, reported whether they experienced a severe headache, mild headache, or no headache each day, and reported how headache(s) interfered in their daily functioning.

#### 2.4.2. Pediatric Migraine Disability Assessment (PedMIDAS)

The PedMIDAS is a brief, paper-and-pencil measure used to assess headache-related disability and burden in youth aged 4 to 18 [19]. It consists of 6 questions that rate disability for the previous three months. Adolescents and their parents are asked how many full and partial days of school were missed due to headaches, how many days the adolescent functioned at less than half of their ability in school due to a headache, how many days the adolescent was not able to do things at home or participate in other activities due to a headache, and for how many days the adolescent participated in other activities but functioned at less than half of their ability due to headaches. These responses are summed to provide a composite score; if a range of days is provided, the high end of the range is used. The composite score indicates disability grade, with 0–10 indicating little to no disability, 11–30 indicating mild disability, 31–50 indicating moderate disability, and greater than 50 indicating severe disability. Adolescents completed this measure with their parents.

#### 2.4.3. Multidimensional Anxiety Scale for Children (MASC)

The MASC is a 39-item, paper-and-pencil self-report measure of anxiety and related symptoms in youth aged 8 to 19 years [20]. The MASC contains four basic scales (physical symptoms, harm avoidance, social anxiety, and separation/panic). Higher total and subscale scores correspond to greater anxiety. *T*-scores of 60–64 are considered slightly elevated, 65–69 elevated, and 70+ very elevated. The measure has excellent psychometric qualities, including excellent internal reliability and satisfactory to excellent test-retest reliability [21].

#### 2.4.4. Center for Epidemiological Studies Depression Scale for Children (CES-DC)

The CES-DC is a 20-item paper-and-pencil self-report measure of depression symptoms in children aged 6 years and older [22]. Youth responded to items about concentration, happiness, appetite, sleep, and mood in terms of how they felt during the previous week before administration of the scale. Each item is rated on a 4-point Likert-type scale from 0 (“not at all”) to 3 (“a lot”). Scores range from 0 to 60, with higher scores indicating greater depressive symptoms, with a score of 15 being indicative of clinically significant depressive symptoms [23].

#### 2.4.5. Chronic Pain Acceptance Questionnaire, Adolescent Version (CPAQ-A)

The CPAQ-A was developed to better understand the processes that contribute to distress and disability in adolescents experiencing chronic pain [24]. The CPAQ-A contains 20 items in a paper-and-pencil self-report format in which adolescents respond to each question on a 5-point Likert-type scale ranging from 0 (“never true”) to 4 (“always true”). Scores range from 0 to 100, with higher scores indicating greater pain acceptance. The adolescent measure contains two subscales. Activity engagement (11 items) is the extent to which adolescents attempt to participate in regular activities while experiencing pain, and pain willingness (9 items) is the extent to which the adolescent describes pain control or reduction as less important than other life goals. Research has found this measure to be highly correlated with disability, depression, anxiety, and confidence in the ability to function normally when in pain [25].

#### 2.4.6. Pediatric Quality of Life Inventory (PedsQL)

The PedsQL is a 23-item paper-and-pencil measure that assesses health-related quality of life in youth aged 2 to 18 years [26]. Both the parent and adolescent self-report versions were used in this study. Subscales include physical functioning (8 items), emotional functioning (5 items), social functioning (5 items), and school functioning (5 items). Respondents rate how much of a problem each item has presented during the past month on a 5-point Likert-type scale ranging from 0 (“never a problem”) to 4 (“almost always a problem”). Scores range as 0–100, with higher scores indicating better health-related quality of life.

### 2.5. Analytic Plan

All data was inspected to ensure normality. In order to reduce the possibility that data from people who dropped out would bias results, we examined data from the complete sample including dropouts and then for individuals who completed treatment; there were no significant differences. Two-tailed paired samples* t*-tests were used to assess mean differences between pretreatment and posttreatment scores on outcome measures.

## 3. Results

### 3.1. Patient Characteristics

The study enrolled 20 adolescent females aged 11–16 years (*M* = 14.15, SD = 1.60, and mode = 15). Participants in the study were mostly Caucasian (94%). Participants were referred to the study via physician referral. Twenty adolescents enrolled in the study. See Table 1 for more demographic details. Of the original 20 adolescents enrolled, 5 (25%) dropped out of the study. One 15-year-old left due to scheduling conflict with extracurricular sports, one 15-year-old left due to severe social anxiety that made her unable to tolerate group treatment, one 11-year-old left due to interpersonal conflict with another group member, and a 16-year-old and 14-year-old left due to academic obligations (e.g., advanced placement classes). All other participants continued to attend classes until the end of the study. However, occasionally participants had to miss a session (attendance range = 12–20 participants) or did not complete diary entries for that week (range = 9–18 completed diaries per week).

### 3.2. Daily Diaries

Participants began recording number of headaches per six-day period between classes at Week 2 (see Table 1 for baseline characteristics). There did not appear to be change in frequency or severity of headaches as reported. Due to a smaller number of participants providing headache diaries in Week 7 (*n* = 8) and Week 8 (*n* = 10), we were unable to conduct analyses to determine empirically whether headache frequency or severity decreased.

Participants reported no adverse events over the course of treatment. Adherence to daily meditation practice was good, with participants reporting an average of 4.69 formal practices per week (SD = 1.84, range = 0–6, and mode = 6 formal practices per week) and most participants reporting regular informal practice. Out of 8 classes, median class attendance was 7 sessions (range = 1–8); mean class attendance was 6.10 sessions (SD = 2.49). Of participants who came at least once after the initial session (*n* = 18), median class attendance was 7 sessions and mean class attendance was 6.67 sessions (SD = 1.88).

### 3.3. Additional Outcome Measures

Participants showed no reduction in headache disability as measured by the PedMIDAS (see Table 2). Participants themselves did not report measurable differences for quality of life as measured by the PedsQL Total Score. However, parents reported increased quality of life as measured by the PedsQL Total Score. Specifically, parents reported improved physical health-related quality of life as measured by the PedsQL Physical Functioning subscale.

Participants reported no reduction in anxiety as measured by the MASC. However, participants reported decreased depressive symptoms on the CES-DC following treatment. On the CPAQ-A, participants showed decreased symptoms on the Pain Withdrawal subscale but not on the Activity Engagement subscale or Total *T*-Score.

### 3.4. Qualitative Data

Teens were provided with open-ended questions in order to elicit participant perspectives on the class in their own words. First, teens were asked, “Did you enjoy participating in the class? Was participating in the class helpful to you?” Of the 15 teens who answered, 8 (53.3%) reported that the class helped by teaching them techniques that helped them cope with stress, relax, and control their emotions and pain. Additionally, 6 participants (40.0%) reported that the class helped in concrete ways, such as decreasing frequency or severity of headaches or helping with sleep or depression. Two participants (13.3%) specifically noted that the class was not as helpful as they had hoped. Finally, 6 participants (40.0%) reported that they enjoyed or loved the class.

Second, participants were asked, “Did participating in this class affect your headaches?” Of the 15 adolescents who responded to this question, 5 (33.3%) said that the class did not affect their headaches. However, 3 (20%) participants stated that the class led to their having fewer headaches, 2 (13.3%) stated that they had less severe headaches as a result of the class, and 1 participant noted that her headaches “got better.”

Finally, participants were asked whether there was anything else that they wanted to tell the group facilitators. Some expressed gratitude, but two responses were particularly striking. One 16-year-old participant stated, “This class was a great part of my week. I liked being in an environment that was so calming and nonjudgmental. I liked that all the girls were going through similar things and therefore I felt more comfortable around them.” Another 16-year-old participant stated, “Thank you so much for providing this class! I'm so glad that I could be a part of it! I've learned skills and techniques that are super helpful. I have never really been around other girls my age who are struggling with the same thing I am and it has been really interesting to hear from them.”

Parents also responded to several open-ended questions. First, parents were asked, “Was participating in this class beneficial for your daughter?” Of 15 parents who responded, 14 (93.3%) responded that the class was beneficial for their daughters. Parents noted a wide variety of beneficial effects, including 3 (20%) who reported that their daughter seemed more calm or relaxed, 2 (13.3%) who said that their daughter coped better with stress, pain, and depression, and 2 (13.3%) who noted that their daughter now got along better with siblings. Other positive effects included keeping up with schoolwork better, sleeping better, focusing at school better, communicating about what bothers her, being more aware of surroundings, and recognizing when things are upsetting her.

Additionally, parents were asked to communicate anything else they wanted to group facilitators. Several parents noted that the class had provided positive benefits for their child beyond headaches. For example, a parent of a 16-year-old said, “I've seen my daughter use breathing techniques to deal with other frustrations and stress besides her pain. She has learned life skills to apply to many situations. Thank you!!” Other parents noted positive interpersonal benefits, particularly their child's ability to be patient with siblings or to communicate about her feelings with her parents.

## 4. Discussion

This study is the first to demonstrate feasibility and acceptability of a group mindfulness-based intervention (MBI) for adolescent girls with recurrent headaches. The primary aim of this study was to evaluate the targeted patient population interest and willingness to commit to the intervention. Of patients who were screened in clinics, some declined to participate citing barriers such as the stressful, obligation-filled time of the school year; the time commitment required for intervention; and geographic barriers. Of the original patients referred to the study by neurologists and other medical providers, it was possible to enroll a large group who were able to attend the scheduled weekly meetings. Given that exclusion criteria for the study were minimal (e.g., no exclusion based on psychiatric comorbidity) and given that the intervention was conducted during a particularly stressful and obligation-filled time during the school year, these results are likely to be generalizable.

On average participants attended six of eight total sessions. Furthermore, participants reported that, on average, they engaged in formal meditation practice 4.69 times out of 6 recorded days each week. At the completion of the intervention, 15 of the original 20 participants were regularly attending weekly sessions. This completion rate of 75% is slightly lower than 89% completion rates observed across other behavioral treatments for recurrent pediatric headaches or migraines [27]. This may have been due to the time of year when the intervention took place (i.e., toward the end of the second semester of the school year) or to differences of adherence in child versus adolescent samples given the higher degree of academic and extracurricular activities occurring during adolescence. Participants who elected to terminate their involvement in the intervention did so for individual reasons not intrinsic to the intervention. Qualitative data indicated that the intervention was positively received by participants, who appreciated the opportunity to learn a behavioral technique to respond to their headaches and related symptoms in more skillful and less judgmental ways. Additionally, participants' parents reported that they would recommend the treatment to other parents of children experiencing chronic pain. Together, this demonstrates that it was possible to recruit and retain participants for a mindfulness-based intervention for headaches and that participant commitment to the intervention was high.

The secondary aim was to evaluate whether the MBI resulted in reduction of headache burden, improved quality of life and acceptance, and reduction of anxiety and/or depression for adolescent females experiencing recurrent headaches. Participants did not report a decrease in headache disability as measured by the PedMIDAS. There are several possible reasons why the intervention did not immediately result in measured decreases in headache disability. First, the participants enrolled in this study reported severe depression and headache burden and may have needed a longer or more intensive course of treatment to experience reduction in headache disability. Indeed, the frequency, amount, or duration of formal mindfulness practice needed to decrease adolescent stress is currently unknown. Although some low-dose intervention studies have found that 20 minutes of meditation per day reduced adult stress and sleep problems [28], necessary dose for adolescents to reduce severe chronic pain and improve quality of life has not yet been quantified. Participants in this study were directed to listen to 10–15-minute guided mindfulness recordings at least once each day or to practice without guidance, and 10–15 minutes per day may not have been adequate to reduce severe headaches. Second, the PedMIDAS measures headache disability over the course of three months, and therefore it may not have been sensitive to changes occurring over the course of an 8-week intervention. Furthermore, it is notable that this group of participants on average reported severe disability as rated by the PedMIDAS, and thus this population may be less likely to see improvement over 8 weeks and require longer duration or more intensive course of treatment.

Regarding quality of life, participants reported that they were better able to accept their pain and were less focused on controlling pain after MBI treatment. Furthermore, parents reported improved quality of life for their child and particularly reported improved physical functioning. This indicates that parents noted improvement in how difficult it was for their adolescent to do things like walk more than one block, run, do sports, lift something heavy, take a bath or shower, or do chores around the house and that they noted their adolescent reporting more energy or less hurt or aches. One explanation is that participants were able to participate more in routine daily activities or complained about aches and pains or low energy with less frequency after treatment. These findings are consistent with prior studies showing that chronic headache patients are less likely to experience pain reduction following MBI intervention than are other populations affected by chronic pain (e.g., back/neck pain patients) and yet still report improved health-related quality of life after MBI treatment [13].

Consistent with previous research, participants in this study reported significant anxiety and depression symptoms. At baseline, 90% of participants self-reported clinically significant depression symptoms and 55% reported elevated anxiety symptoms. On average, participants reported a significant reduction in symptoms of depression following the intervention, although CES-DC scores indicate that participants remained clinically depressed following treatment. This reduction is consistent with a recent meta-analysis of studies evaluating MBI treatments for mood symptoms that demonstrated that such interventions have robust effects for adults [29] and is also consistent with recent studies showing that MBI reduces depressive symptoms for adolescent outpatients [30]. Further examination of the effects of MBI in headache and comorbid anxiety and depression would be clinically useful.

Finally, participants described mostly positive experiences with the intervention via qualitative data. Teens' qualitative reports echoed and expanded upon the quantitative data by reflecting that while teens did not experience a decrease in headache frequency or severity, they found the intervention helpful in regaining a feeling of control over their pain or emotional responses to pain and in normalizing the experience of headaches. Overall, parents overwhelmingly reported that the intervention was beneficial in helping participants relax and cope better with pain and depression and in improving relationships with siblings and parents.

This study had several limitations that warrant mention. First, given that several participants noted the positive benefits of interacting with other adolescents who also struggled with recurrent headaches, a randomized controlled trial with a supportive psychotherapy control group would help determine whether mindfulness is beneficial for quality of life over and above the normalization provided by group treatment. Second, future research should incorporate measures of adolescent mindfulness [31] and stress/headache mechanisms [32]. Third, it will be important for studies to incorporate baseline daily diary collection four or more weeks before treatment begins and to further strategize how to ensure more diligent completion of the diary by participants. Electronic reminders or an electronic daily diary to be completed on cell phones or computers might increase headache and MBI practice reporting among adolescents. Fourth, this study recruited only adolescent females with recurrent headaches, and the results may not generalize to adolescent males with recurrent headaches. Finally, an important limitation is the lack of follow-up data for these participants. One-month follow-up occurred at the end of the school year, and very few participants responded to requests for follow-up data at that time. Thus an important future direction for research is whether adolescents continue their high adherence to formal mindfulness practice after ceasing to participate in structured group MBI treatment.

To conclude, this pilot study demonstrated that it is feasible to recruit a large adolescent sample for MBI treatment for headaches and potentially for other chronic pain conditions as well. Participants reported that the treatment was acceptable and helped them feel more in control of their pain. Additionally, MBI reduced depression and improved quality of life with no adverse effects, making group-based MBI a potentially beneficial behavioral adjunct treatment for adolescents suffering from recurrent headaches.

## Figures and Tables

**Table 1:** Demographic and baseline characteristics (*N* = 20).

	% (*n*)	Range	Mean (SD^a^)
Age	—	11–16 years	14.15 (1.60)
Baseline headaches	—	0–6 headaches	4.6 (1.80)
Baseline severe headaches	—	0–6 headaches	2.6 (1.96)
Headache disability			
PedMIDAS^b^	—	3–270	66.95 (67.45)
Little or no disability (0–10)	10 (2)	—	—
Mild disability (11–30)	25 (5)	—	—
Moderate disability (31–50)	15 (3)	—	—
Severe disability (50+)	50 (10)	—	—
Quality of life			
Teen PedsQL^c^ Total	—	27.17–88.04	55.65 (15.54)
Teen PedsQL Physical Functioning	—	28.13–93.75	56.88 (17.81)
Teen PedsQL Psychosocial Functioning	—	20–91.67	55.00 (18.20)
Parent PedsQL Total	—	30.43–92.39	57.83 (16.79)
Parent PedsQL Physical Functioning	—	28.13–100	58.13 (19.49)
Parent PedsQL Psychosocial Functioning	—	30–96.67	57.67 (16.97)
Pain acceptance			
CPAQ-A^d^ Activity Engagement	—	2–40	26.20 (9.85)
CPAQ-A Pain Withdrawal	—	6–35	20.45 (7.56)
CPAQ-A Total	—	24–59	46.65 (8.02)
Depression			
CES-DC^e^ Total	—	9–51	31.20 (13.04)
Not clinically significant	10 (2)	—	—
Clinically significant (16+)	90 (18)	—	—
Anxiety			
MASC^f^ Total	—	42–71	56.20 (9.47)
Low (<40)	0 (0)	—	—
Average (40–54)	40 (8)	—	—
High average (55–59)	15 (3)	—	—
Slightly elevated (60–64)	25 (5)	—	—
Elevated (65–69)	5 (1)	—	—
Very elevated (70+)	10 (2)	—	—

^a^SD: standard deviation; ^b^Pediatric Migraine Disability Assessment; ^c^Pediatric Quality of Life Inventory; ^d^Chronic Pain Acceptance Questionnaire, Adolescent Version; ^e^Center for Epidemiological Studies Depression Scale for Children; ^f^Multidimensional Anxiety Scale for Children.

**Table 2:** Pre- and posttreatment outcome measures for treatment completers (*N* = 15).

	Pretreatment mean (SD^a^)	Posttreatment mean (SD)	Pre/postcomparison
Headache disability			
PedMIDAS^b^	62.07 (64.66)	57.33 (73.90)	*t*(14) = .554, *p* = .589
Quality of life			
Teen PedsQL^c^ Total	53.26 (10.32)	58.19 (11.90)	*t*(14) = −1.388, *p* = .187
Teen PedsQL Physical Functioning	56.04 (15.73)	60.63 (19.22)	*t*(14) = −.743, *p* = .470
Teen PedsQL Psychosocial Functioning	51.78 (11.88)	56.89 (9.82)	*t*(14) = −1.440, *p* = .172
Parent PedsQL Total	55.87 (13.90)	63.33 (13.06)	*t*(14) = −2.157, *p* = .049^*∗*^
Parent PedsQL Physical Functioning	56.25 (18.18)	66.88 (3.93)	*t*(14) = −2.377, *p* = .032^*∗*^
Parent PedsQL Psychosocial Functioning	55.67 (13.60)	61.44 (13.76)	*t*(14) = −1.632, *p* = .125
Pain acceptance			
CPAQ-A^d^ Activity Engagement	25.40 (8.74)	28.40 (9.49)	*t*(14) = −1.695, *p* = .112
CPAQ-A Pain Withdrawal	22.47 (6.46)	17.47 (5.18)	*t*(14) = 3.293, *p* = .005^*∗*^
CPAQ-A Total	47.87 (6.95)	45.87 (9.08)	*t*(14) = 1.088, *p* = .295
Depression			
CES-DC^e^ Total	33.33 (11.54)	26.07 (12.60)	*t*(14) = 3.034, *p* = .009^*∗*^
Anxiety			
MASC^f^ Total	58.29 (8.56)	56.29 (9.11)	*t*(13) = 1.038, *p* = .318

*∗* indicates statistically significant differences in two-tailed pre/postpaired samples *t*-tests.

^a^SD: standard deviation; ^b^Pediatric Migraine Disability Assessment; ^c^Pediatric Quality of Life Inventory; ^d^Chronic Pain Acceptance Questionnaire, Adolescent Version; ^e^Center for Epidemiological Studies Depression Scale for Children; ^f^Multidimensional Anxiety Scale for Children.

## Abbreviations

MBI: Mindfulness-based intervention
MBSR: Mindfulness-based stress reduction
MBCT: Mindfulness-based cognitive therapy
PedMIDAS: Pediatric Migraine Disability Assessment
MASC: Multidimensional Anxiety Scale for Children
CES-DC: Center for Epidemiological Studies Depression Scale for Children
CPAQ-A: Chronic Pain Acceptance Questionnaire, Adolescent Version
PedsQL: Pediatric Quality of Life Inventory.
